# Pig pangenome graph reveals functional features of non-reference sequences

**DOI:** 10.1186/s40104-023-00984-4

**Published:** 2024-02-22

**Authors:** Jian Miao, Xingyu Wei, Caiyun Cao, Jiabao Sun, Yuejin Xu, Zhe Zhang, Qishan Wang, Yuchun Pan, Zhen Wang

**Affiliations:** 1 College of Animal Sciences, Zhejiang University, Hangzhou, 310058 Zhejiang China; 2 Yazhou Bay Science and Technology City, Hainan Institute of Zhejiang University, Yazhou District, Building 11, Yongyou Industrial Park, Sanya, 572025 Hainan China

**Keywords:** Heat tolerance, Immune ability, Non-reference sequences, Pig pangenome

## Abstract

**Background:**

The reliance on a solitary linear reference genome has imposed a significant constraint on our comprehensive understanding of genetic variation in animals. This constraint is particularly pronounced for non-reference sequences (NRSs), which have not been extensively studied.

**Results:**

In this study, we constructed a pig pangenome graph using 21 pig assemblies and identified 23,831 NRSs with a total length of 105 Mb. Our findings revealed that NRSs were more prevalent in breeds exhibiting greater genetic divergence from the reference genome. Furthermore, we observed that NRSs were rarely found within coding sequences, while NRS insertions were enriched in immune-related Gene Ontology terms. Notably, our investigation also unveiled a close association between novel genes and the immune capacity of pigs. We observed substantial differences in terms of frequencies of NRSs between Eastern and Western pigs, and the heat-resistant pigs exhibited a substantial number of NRS insertions in an 11.6 Mb interval on chromosome X. Additionally, we discovered a 665 bp insertion in the fourth intron of the *TNFRSF19* gene that may be associated with the ability of heat tolerance in Southern Chinese pigs.

**Conclusions:**

Our findings demonstrate the potential of a graph genome approach to reveal important functional features of NRSs in pig populations.

**Supplementary Information:**

The online version contains supplementary material available at 10.1186/s40104-023-00984-4.

## Background

Pigs (*Sus scrofa*) are crucial to human society, serving as a valuable source of animal protein and holding immeasurable medical potential due to their similarities with humans in terms of genomics, anatomy, and physiology [1]. Genomic data processing in pigs primarily begins with read mapping to the pig reference genome (*Sus scrofa 11.1*), which is assembled from a female European Duroc pig. However, relying solely on a single reference genome fails to capture the full genomic diversity of the species, particularly considering pigs' independent domestication history spanning over 9,000 years in Europe and Asia [2]. Consequently, utilizing a single pig assembly as the reference genome introduces biases in read mapping and hinders genetic research on pigs by constraining the accurate identification of genetic variants [3].

The concept of the pangenome offers a solution to overcome the limitations of a single reference genome. A pangenome refers to a comprehensive collection of genomic sequences within a species or a specific phylogenetic clade, capturing a broader spectrum of genetic diversity [4]. The NRSs, referring to DNA sequences missing from a certain reference genome, have potential effects on economic traits or diseases by disrupting genomic regions of exons or regulatory elements [5, 6]. For instance, the pig pangenome (PIGPAN) has been recently constructed by using 12 assemblies of Eurasian pigs [7, 8]. The NRSs were identified by aligning contigs from 11 assemblies to the *Sus scrofa 11.1* and regarded as unplaced contigs to extend the *Sus scrofa 11.1*. Through comparing the frequencies of NRSs in Chinese pigs and European pigs, they discovered a prevalent NRS in Chinese pigs containing the complete genic region of the tazarotene-induced gene 3 (*TIG3*) gene, which is involved in fatty acid metabolism [8].

“NRSs + reference genome” is commonly employed to construct the liner format of a pangenome [9, 10]. However, accurately placing NRSs on the reference genome remains a challenge, as only a small proportion (e.g., 11,028 out of 39,744, 27.7% for pig [8]; 546 out of 18,231, 29.98% for cattle [11]) of NRSs can be successfully positioned on the reference genome. This suggests that the challenge of accurately positioning NRSs on the reference genome persists as a significant obstacle. The unplaced NRSs lack a stable coordinate system, making them less useful for other researchers. Therefore, additional efforts are required to enhance the presentation of position information in the linear format of pangenomes.

The recent development of graph-based genome structures has enabled the construction of pangenome graphs [12, 13]. Compared to a linear reference genome, the genome graph is capable of representing the genetic information from diverse breeds within a species, thereby reducing mapping bias and increasing sensitivity in detecting variants, particularly structural variants [14–16]. Typically, a pangenome graph establishes the reference genome as the backbone, thus preserving the coordinate system of the reference genome. The fundamental units of a graph genome are nodes, which represent sequence segments interconnected by edges (also known as links) that depict the relationships between various sequence segments [13]. Pangenome graphs have been well applied to human and plant genomes [14, 17–20], but their utilization on farm animals (except cattle) is still few [11, 21–23]. To date, we have only found one study reporting the pig pangenome graph. However, their study primarily focused on structural variant breakpoints rather than novel sequences [24].

Studying the genetic mechanisms of animals in extreme environments is of great importance for understanding their evolution, gaining insights into their unique physiological and biochemical mechanisms, which can contribute to medical advancements, as well as predicting species' responses to future environmental changes for conservation purposes [25–27]. However, previous research on the environmental adaptation of domestic animals has predominantly focused on single nucleotide polymorphisms (SNPs), with limited investigation into non-reference sequences (NRSs).

In this study, we constructed a pig pangenome graph by integrating two de novo assemblies with 18 publicly available assemblies. Through this approach, we identified 23,831 high-quality NRSs, totaling 105 Mb in length, which are absent from the autosomes and sex chromosomes of the *Sus scrofa 11.1* genome. Additionally, we conducted functional annotation analysis and investigated the potential biological functions of these NRSs by exploring their association with economic traits and environmental adaptation. Our findings also highlight that integrating NRSs into the reference genome can significantly enhance the mapping quality of the pig genome.

## Materials and methods

### De novo assembly of two Chinese local pig breeds

We collected whole blood from two representative male individuals, including SWT (Shawutou, Shanghai, China) and TC (Tunchang, Hainan, China) pig breeds. Genomic DNA was extracted from blood using QIAGEN^®^ Genomic kit. The extracted DNA was evaluated for degradation and contamination using 0.75% agarose gels. DNA purity was assessed using a NanoDrop^TM^ One UV–Vis spectrophotometer (Thermo Fisher Scientific, Waltham, Massachusetts, USA), with OD_260/280_ ratios ranging from 1.8 to 2.0 and OD_260/230_ ratios ranging from 2.0 to 2.2. Subsequently, the DNA concentration was measured using a Qubit^®^ 3.0 Fluorometer (Invitrogen, Carlsbad, California, USA). A total of 2 µg of DNA was used for library preparations. To prepare the library, the genomic DNA sample was sheared into fragments of the expected size using g-TUBEs (Covaris, Woburn, Massachusetts, USA). Single-strand overhangs were then removed, and DNA fragments underwent damage repair, end polishing, and ligation with the stem-loop adaptor for PacBio sequencing. Link-failed fragments were eliminated using exonuclease, and the target fragments were screened using the BluePippin system (Sage Science, Beverly, Massachusetts, USA). The SMRTbell library was subsequently purified using AMPure PB, and the size of library fragments was assessed using the Agilent 2100 Bioanalyzer (Agilent Technologies, Santa Clara, California, USA). The sequencing process was performed on a PacBio Sequel II platform. The raw Circular Consensus Sequencing (CCS) reads were processed using the ccs software (–min-passes 1 –min-rq 0.99 –min-length 100) to eliminate low-quality reads and adapters."

The primary contig assembly was generated using high-quality HiFi reads by Hifiasm (version 0.16.1-r375) [28] with default parameters. The completeness of the assembled contigs was evaluated using BUSCO (Benchmarking Universal Single-Copy Orthologs, version 5.43) [29] based on mammalian single-copy homologous gene database (mammalia_odb10.2021-02-19). The contigs were then anchored to chromosomes using Ragtag (version 2.1.0) [30] guided by the pig reference genome [31] and the chromosome-level genomes were annotated using liftoff [32]. NUCmer application of the MUMmer software (version 4.0.0) [33] was employed to align each of the two genomes against the reference genome. The alignments of homologous DNA sequences were visualized using RIdeogram (version 0.2.2) [34].

### Variants discovery from the two novel assemblies

The contigs from the two new pig assemblies were aligned to the *Sus scrofa 11.1* using minimap2 [35], and variants were identified using minimap2 module paftools.js based on the assembly-versus-assembly alignment. We annotated the variants using the Ensembl Variant Effect Predictor (VEP) build 104 [36] and identified deleterious variants using SIFT [37]. Genes harboring deleterious variants were extracted to perform enrichment analysis using KOBAS [38].

### Construction of pig pangenome graph

A total of 21 pig assemblies (Table 1), including the reference genome, two de novo assemblies generated in this study, and 18 published assemblies, were used to construct the pangenome graph using minigraph (version 0.17-r524) [12]. The detailed procedures are described below. For the 13 pig genomes assembled with only short sequencing reads, we firstly corrected the potential mis-assemblies and anchored the corrected contigs or scaffolds to chromosome level using Ragtag. We then estimated the assembly-wise genetic distance between the reference genome with the remaining 20 genomes using Mash [39]. Finally, taking the *Sus scrofa 11.1* as the backbone of the pangenome graph, we progressively incorporated other assemblies into the backbone in the order of increasing genetic distance from *Sus scrofa 11.1* using minigraph. To simplify the graph structure, only autosomes and sex chromosomes were considered when pangenome generation. A phylogenetic tree was built using MegaX [40] with assembly-wise genetic distance as input and then visualized by iTOL [41].

**Table 1:** Details of 21 pig genome used for construction of pig pangenome graph

Genome	Breed	Sex	Assembly level	GenBank accession	Region	Reference
*Sus scrofa 11.1*	Duroc	Female	Reference	GCA_000003025.6	Europe	[31]
USMARC	US crossbreed pig	Male	Chromosome	GCA_002844635.1	America	
DU	Duroc	Male	Chromosome	GCA_015776825.1	Europe	[51]
MS	Meishan	Female	Chromosome	GCA_017957985.1	Asia	[52]
NX	Ningxiang	Female	Chromosome	GCA_020567905.1	Asia	[53]
BMmini	Bamaxiang	Male	Chromosome	GCA_007644095.1	Asia	[54]
HP	Hampshire	Female	Scaffold	GCA_001700165.1	Europe	[7]
LR	Landrace	Female	Scaffold	GCA_001700215.1	Europe	
BK	Berkshire	Female	Scaffold	GCA_001700575.1	Europe	
PT	Pietrain	Female	Scaffold	GCA_001700255.1	Europe	
LW	Large White	Female	Scaffold	GCA_001700135.1	Europe	
BM	Bamei	Female	Scaffold	GCA_001700235.1	Asia	
RC	Rongchang	Female	Scaffold	GCA_001700155.1	Asia	
TB	Tibetan	Female	Scaffold	GCA_000472085.2	Asia	
JH	Jinhua	Female	Scaffold	GCA_001700295.1	Asia	
WZS	Wuzhishan	Male	Scaffold	GCA_000325925.2	Asia	[55]
EUW	European wild boar	Male	Scaffold	GCA_021656055.1	Europe	[56]
KNY	Kenya domestic pig	Male	Scaffold	GCA_019290145.1	Africa	[57]
EGM	Ellegaard Gottingen minipig	Female	Scaffold	GCA_000331475.1	Europe	-
SWT	Shawutou	Male	Contig	-	Asia	-
TC	Tunchang	Male	Contig	-	Asia	-

### Detection of NRSs

During the construction of the pig pangenome graph via Minigraph, nodes were labelled automatically if they were sourced from the reference genome. To validate the accuracy of node labeling, we performed individual genome realignment to the graph using Minigraph with the “-cxasm –call” option. Following this, node labelling was conducted again based on the respective paths of each genome. Nodes that failed to appear in any genome or exhibited inconsistent labelling were considered as skeptical nodes and subsequently excluded from the analysis. After removing skeptical nodes, the remaining nodes in the pig pangenome graph that are not originating from the reference genome were regarded as non-reference nodes (NRNs). Then, the paths were considered as NRSs following the below criteria: 1) present in at least one assembly; 2) contain at least one NRN; 3) have a length greater than 500 bp; and 4) have the cumulative length of NRNs represents over 50% of the total length of the NRS. Next, we removed redundant NRSs by all-vs-all BLAST [42]. Briefly, we built a BLAST database using all NRSs and then aligned all NRSs against the database. We removed the shorter NRS in a high-scoring segment pairs (≥ 90% identity and ≥ 90% coverage), and then aligned the remaining NRSs to the *Sus scrofa 11.1*. The NRSs that failed to align to the *Sus scrofa 11.1* were classified into cNRSs (complete NRSs) and pNRSs (partial NRSs) according to whether they contained the reference nodes.

### Functional characterization of NRSs

Enrichment analyses were performed to investigate the biological functions of the NRSs. We used clusterprofiler [43] to carry out Gene Ontology (GO) enrichment analysis for genes whose exon or CDS regions were affected by the NRS insertion events. We set 0.2 as the significant threshold for the *P*-values adjusted by “Benjamini-Hochberg” method [44]. To explore the impact of these NRS insertion events on economic traits, we downloaded pig QTL database from animal QTL database [45] and then extracted the QTL where the NRS insertion occurred. The QTL enrichment analyses were performed using GALLO [46] and the trait with adjusted *P* value < 0.01 was considered as significant.

### Discovery of the presence/absence variation of NRSs (PAV-NRSs)

We downloaded 192 pig genome resequencing samples with an average depth of 21, representing 14 different pig breeds with sample sizes ranging from 5 to 57. The PAV-NRSs detection for each sample was carried out as following steps: 1) Extraction of unmapped reads and reads with mapping quality < 10. The WGS data of 192 individuals has already been mapped to the *Sus scrofa 11.1*. using BWA in our previous study [47]. 2) Mapping extracted reads to the NRSs. We used the pair-end and single-end mapping models for paired reads and single reads extracted in step 1, respectively. Besides, each NRS was added with a 100 bp flank sequence to facilitate the mapping processes [21]. The mapping results from the pair-end and single-end models were merged using samtools [48]. 3) Calculation of NRS coverage using mosdepth [49]. If an NRS had coverage of more than 70% of its length, it was considered present in the given sample.

The NRSs were then categorized based on their presence frequencies, with those present in more than 99% of the individuals defined as core NRSs, those present in 90%–99% of individuals defined as softcore NRSs, those present in 1%–99% of individuals defined as shell NRSs, and those present in less than 1% of individuals defined as cloud NRSs. NRSs that did not occur in any individual were defined as skeptical NRSs and were not retained for presence/absence variation (PAV) analysis or gene annotation. We generated a PAV matrix that records whether an NRS is present in a sample, and performed principal component analysis (PCA) of the PAV matrix using the R function *eigen*.

### Identification of the associations between NRSs and pig environmental adaptation

To further reveal potential associations of NRSs with pig environmental adaptation, we conducted a population-scale PAV analysis of NRSs. Here, we used 192 whole genome sequencing (WGS) data (see details in Table S1) to calculate frequencies of NRSs in three comparisons: European pigs vs. Asian pigs, heat-resistant pigs vs. cold-resistant pigs, and high-altitude pigs vs. low-altitude pigs. Subsequently, we employed the Fisher's exact test to compare the differences in NRS frequencies among the six groups in these three comparisons, with the resulting* P*-values adjusted using Bonferroni correction. NRSs were considered significantly different if the adjusted *P* values threshold was below 0.01. Genes affected by significant NRS insertions (overlapped with significant NRSs) were extracted from the ensemble Gene annotation file (Sus_scrofa.Sscrofa11.1.107.gtf). The corresponding Entrez IDs were obtained by transforming from the gene symbols using R package biomart [50]. The GO enrichment analysis was carried out based on entrez IDs by R package clusterprofiler to detect the function of the affected genes.

### Prediction of gene structure in NRSs

To identify the novel genes embedded in NRSs, the de novo and homology strategies were utilized to identify repeats within NRSs first. A de novo repeat library was built by scanning the NRSs using RepeatModeler [58]. The homologous repeat annotation library was constructed by extracting mammalian repeats sequences from the combined library comprising Repbase (release 20181026) and Dfam (version 3.2) [59]. Our custom library and homologous library were passed together to RepeatMasker [60] to mask repeats. MAKER3 pipeline [61] was applied to carry out the prediction of the gene structures in the masked NRSs. To generate the predicted gene structures, MAKER3 combined three distinct evidences, including ab initio prediction, cDNA and homologous proteins. Augustus [62] was used to perform ab initio prediction using human model. The cDNA sequences and protein sequences from pigs and 11 related mammals (*Homo sapiens*, *Equus caballus*, *Canis lupus*, *Bos_taurus*, *Capra_hircus*, *Ovis_aries*, *Camelus dromedaries*, *Delphinapterus leucas*, *Balaenoptera musculus*, *Physeter catodon*, and *Tursiops truncatus*) were downloaded from Ensembl release 106. Two rounds all-versus-all BLAST (identity > 90% and coverage > 90%) were used to remove the redundant cDNA and protein sequences. The non-redundant cDNA sequences and protein sequences were treated as evidences of transcribed RNA and homologous proteins, respectively. Finally, only predicted gene models with annotation edit distance values smaller than 0.5 and amino acid numbers larger than 50 were retained.

### Identification of high-quality novel genes in NRSs

We first removed the redundant predicted gene models (identity value ≥ 0.8) using CD-HIT [63]. Next, we aligned the protein sequences of the remaining predicted gene models to the pig reference genome using BLAST, and then labeled gene models aligned to reference genome as ‘reference-like genes’. Finally, the gene models were labeled as ‘repeat-related genes’ if they have more than 50% of repeat sequences. The predicted gene models after removing redundant genes, reference-like genes, and repeat-related genes were regarded as high-quality novel genes. To obtain the putative gene symbols and GO terms, functional annotation of the high-quality novel genes was carried out using InterProScan (version 5.56–89.0) [64], Swissprot and KOBAS. Considering that the human genome is more well-annotated than pig genome, the protein sequences of high-quality novel genes were also submitted to KOBAS to perform enrichment analysis based on 5 databases of GO, KEGG pathway, KEGG disease, OMIM and GWAS catalog, while human was set as the species.

### The expression of high-quality novel predicted genes

A total of 92 raw RNA-seq data of different tissue from 10 different pig breeds (Berkshire, Hampshire, Landrace, Large White, Pietrain, Bamei, Jinhua, Meishan, Rongchang and Tibetan) were downloaded from NCBI short-read archive database (projectID PRJNA311523) and 53 raw RNA-seq data of different tissues from a Chinese female Bamaxiang pig were downloaded from CNSA (China National GenBank database Sequence Archive) (projectID CNP0001361). Therefore, a total of 145 raw RNA-seq data from 11 different pigs were collected from public datasets. To verify the novel predicted genes as ‘real’ genes, we examined predicted novel gene expression profiles from RNA-seq data. We removed low-quality reads and adapters in the raw RNA-seq reads using fastp [65] and then mapped the remaining reads to the NRSs containing high-quality novel genes by Hisat2 [66]. Next, transcripts were assembled and quantified using StringTie (v.2.1.7) [67] guided by the *Sus scrofa 11.1*. We evaluated the TPM of each transcript and TPM > 0 is used to determine whether the transcript is present in a sample. If one certain transcript occurred in at least one sample, we considered this transcript as validated.

## Results

### De novo assembly of two Chinese local pig breeds

**Fig. 1:**
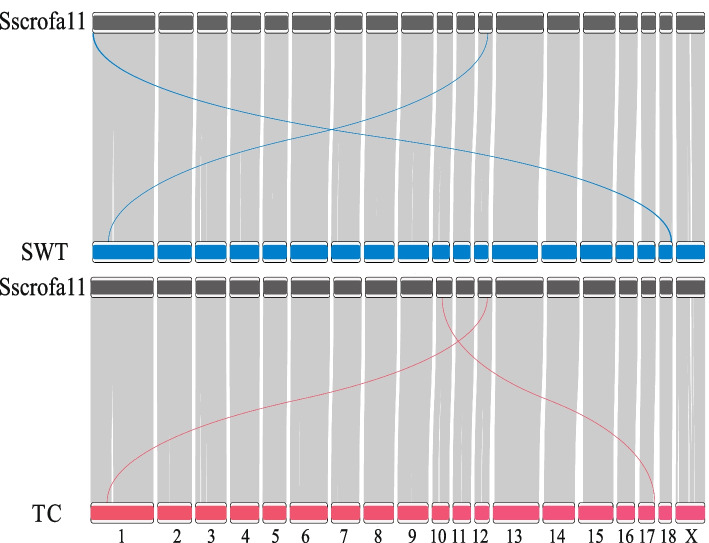
Visualization of the genome synteny of *Sus scrofa 11.1* (Sscrofa11) assembly with Shawutou (SWT) assembly and Tunchang (TC) assembly

The genomic sequences of two local Chinese pig breeds, Shawutou (SWT) and Tunchang (TC), were obtained with an approximate coverage depth of 36X and 26X, respectively. The two de novo assemblies consisted of 169 and 216 contigs with a contig N50 length of 77.3 Mb and 64.7 Mb, and had 96.15% and 96.36% BUSCO completeness scores respectively (see assembly details in Table 2). Compared with the recently assembled genomes of Ningxiang pig (418 contigs; N50 = 26.1 Mb), Meishan pig (1,430 contigs; N50 = 33.65 Mb), the genomes of TC pig and SWT pig achieve a comparable quality (Fig. S1). The collinearity analysis showed the two de novo genomes have a high collinearity with the reference genome, expect two inter-chromosomal alignments (Fig. 1). An inter-chromosomal alignment with close physical proximity occurred in each genome: A sequence spanning from 41.12 to 41.20 Mb on chromosome 12 of the SWT pig genome, aligns with a region from 75.12 to 75.04 Mb on chromosome 1 of the reference genome. Similarly, in the TC pig genome, a segment from 41.11 to 41.20 Mb on chromosome 12 aligns with a region from 76.16 to 76.07 Mb on chromosome 1 of the reference genome.

**Table 2:** De novo assembly of two Chinese local pigs

Breed	Raw bases, Gb	Depth	Assembly length, Gb	Contig number	Contig N50, Mb	BUSCO
SWT	96.05	35.97	2.68	169	77.25	96.15%
TC	70.43	26.28	2.67	216	64.72	96.36%

### Variants discovery from the two novel assemblies

In total, we identified over 15 million variants in the genomes of SWT and TC pigs. Specifically, these variants encompassed 12,367,163 and 12,483,441 SNPs, 3,057,303 and 3,064,290 INDELs, as well as 62,636 and 62,563 structure variants, respectively. The VEP revealed a similar distribution of coding variants in SWT and TC pigs, with approximately 62% are synonymous, 32% are missense and 4% are frameshift mutations. A total of 2,082 and 1,995 genes are affected by deleterious variants for SWT and TC pigs, respectively, with only 1,125 genes were common. The enrichment analysis of affected genes shows that 13 and 12 significant (adjusted *P* value < 0.05) KEGG pathways were identified for SWT and TC pigs, among which 8 KEGG pathways were common (Table S2). Additionally, a total of 68 significant GO terms were identified for either SWT or TC pigs (Table S2).

### Construction of the pig pangenome graph

We constructed the pig graph genome using 21 curated pig assemblies from 20 pig breeds, including 10 Asian domestic breeds, 7 European breeds, 2 crossbreds and 1 African breed (Table 1). First, we anchored the 15 genomes (13 publicly available genomes and 2 genomes assembled in this study) that were not assembled at chromosome level to chromosome, with an average of 96.68% anchoring rates ranging from 95.72% to 97.94% (Table S3). We then estimated the assembly-wise genetic distances using Mash (Table S4) and used them to construct a phylogenetic tree (Fig. S2). We found these pig breeds majorly clustered into three groups: (1) 2 miniature pigs (WZS and EGM); (2) 9 Asian pig breeds, and (3) 8 European pigs, 1 African pig, and the US crossbred USMARC pig.

The *Sus scrofa 11.1* was set as the backbone of the graph, with the remaining 20 genomes added incrementally according to their genetic distance to *Sus scrofa 11.1*. The pig pangenome graph consisted of 999,426 nodes linked by 1,415,311 edges, integrating a total of 2,581,065,767 bases, with 94.35% (2,435,262,063) originating from the reference genome. In comparison to the previously published pig pangenome (552,018 nodes, 664,789 edges and 2,705,225,506 bases) [24], our pangenome contains fewer bases, while featuring a higher count of nodes and edges. The increased number of nodes and edges in our pan-genome can be attributed to the incorporation of a larger number of diverse pig breeds, resulting in increased genetic variants and complexity within the pangenome. Conversely, the reduced number of bases in our pan-genome can be primarily ascribed to our deliberate focus on including only autosomes and sex chromosomes.

### Detection of NRSs

**Fig. 2:**
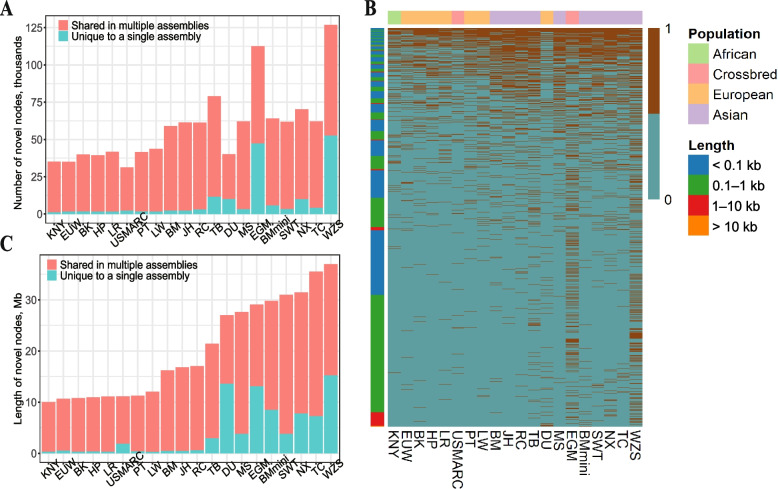
Distribution of non-reference nodes (NRNs). **A** and **B** Total length and number of NRNs in specific assemblies or shared by multiple assemblies. **C** Heatmap of the presence/absence variation (PAV) of NRNs within 20 assemblies, with colored blocks on the left representing different lengths of NRNs and blocks on top representing different pig populations

After removing 4,453 skeptical nodes, a total of 348,770 credible non-reference nodes (with a cumulative length of 144 Mb) were identified. Among them, 812 NRNs were present in all 20 non-reference genomes, while 171,660 NRNs were present in only one genome (Table S5). Notably, Eastern pigs possessed a higher number of NRNs (ranging from 59,015 to 126,932) than Western pigs (ranging from 35,048 to 43,690) (Fig. 2A and B). In particular, the miniature pigs (WZS, EGM) exhibited a greater quantity and cumulative length of NRNs in comparison to other pigs. The WZS genome supported the largest number of NRNs (126,932) with a total cumulative length of 30.93 Mb (Fig. 2C). In general, the number of NRNs from a given genome was found to increase as the genetic distance from the reference genome increased. We also attempted to analyze the relationship between gender and NRN lengths. We found that although the average NRN length in boars was greater than in sows, the overall difference was not statistically significant (Fig. S3).

**Fig. 3:**
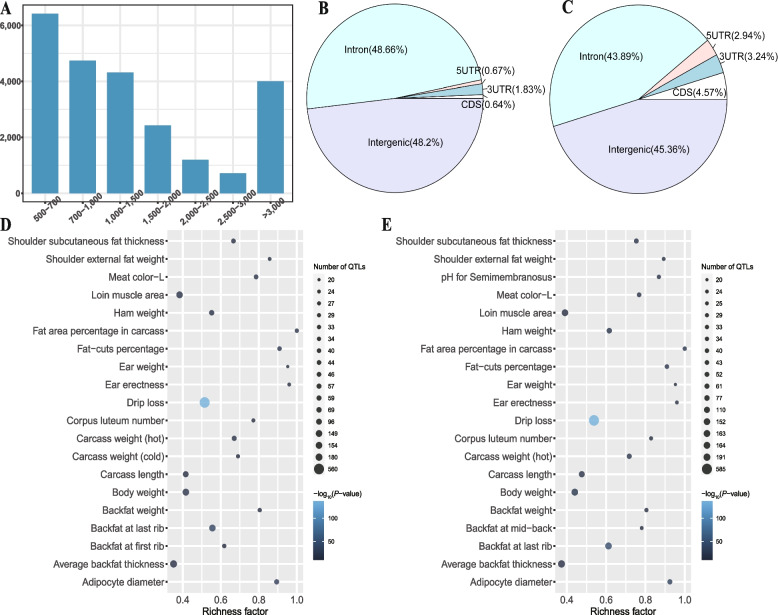
Characterization of non-reference sequences (NRSs). **A** Length distribution of NRSs. **B** and **C** Proportions of cNRSs (**B**) and pNRSs (**C**) in 5'UTR, 3'UTR, CDS, introns, and intergenic regions. **D** and **E** Bubble plots of QTL enrichment analysis on QTLs affected by cNRSs (**D**) and pNRSs (**E**)

After filtering out redundant NRSs (7,866) and reference-like NRSs (10,231) from the 41,928 raw NRSs, we identified 23,831 high-quality NRSs with a total length of 105.16 Mb (Table S6). The high-quality NRSs had an N50 of 29.97 kb, but only 16.81% of them had a length larger than 3 kb (Fig. 3A). We classified NRSs into 8,060 cNRSs and 15,771 pNRSs according to whether they contained reference nodes. We found that these NRSs evenly distributed across all chromosomes except for the Y chromosome, which has fewer NRS insertion events (Fig. S4). The observed reduction in the number of NRSs on the Y chromosome could be attributed to the limited representation of male samples. Moreover, the majority of NRS insertion events occurred in the genomic region of intergenic (46.27%) and intron (45.42%), and a smaller proportion of them located in CDS regions (0.64% for cNRSs and 4.57% for pNRSs, Fig. 3B and C).

### Functional features of NRSs

A total of 35 and 356 unique genes whose CDS regions overlapped with cNRS and pNRS insertions, respectively. The GO enrichment analysis revealed that cNRS-affected genes were not significantly enriched in any GO terms with an adjusted *P* value ≤ 0.2. In contrast, pNRS-affected genes significantly enriched in five GO terms. Specifically, three biological processes were enriched: antimicrobial humoral response (adjusted *P* = 0.02), defense response to bacterium (adjusted *P* = 0.096), and antibacterial humoral response (adjusted* P* = 0.183). Additionally, two molecular functions were enriched: metallopeptidase activity (adjusted *P* = 0.138) and olfactory receptor activity (adjusted *P* = 0.151) (Table S7). The GO enrichment analysis results imply that pNRS insertion events may impact the biological processes and molecular functions of genes related to immunity reinforcement, such as *ACP5*, *PI3*, *DEFB1*, *LTF*, *WFDC2*, *PR39*, *SPAI-2*, *NPG4* and *NPG1*.

The cNRS and pNRS insertions showed similar overlapping proportions in different economic traits (Fig. S5, S6), the majority of them (approximately 65%) located in genomic regions related to meat and carcass QTLs. A total of 146 and 143 economic traits were significantly enriched for cNRSs and pNRSs (Table S8, S9), and the top enriched QTL was drip loss (Fig. 3D and E).

### Discovery of the PAV-NRSs (presence/absence variation of NRSs)

**Fig. 4:**
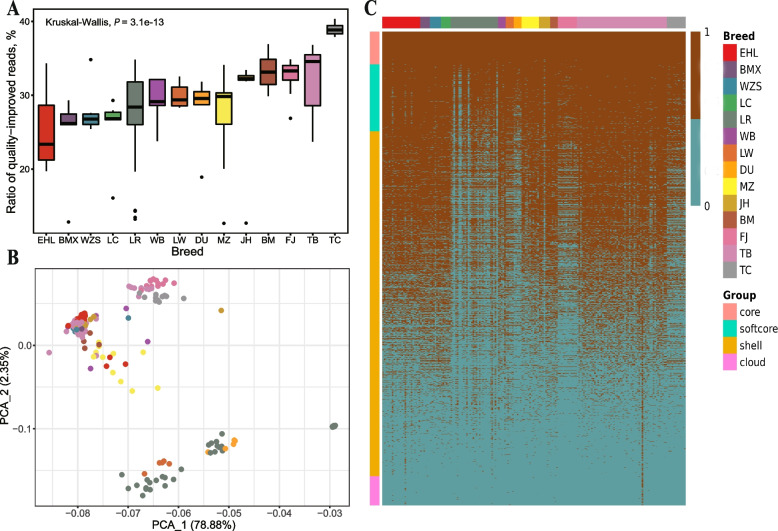
Evaluation of the population pattern of NRSs using 192 WGS data. **A** Ration of reads with improved mapping quality when NRSs were taken into consideration. **B** PCA plot using NRSs. **C** Heatmap showing the PAV of NRSs in a population scale

On average, approximately 30% of unmapped and low-quality mapped reads obtained a higher mapping quality (MQ > 10) when mapping to the NRSs (Table S10). The ratios of quality-improved reads varied significantly among different breeds (*P* = 1.8e-13) and the TC pigs exhibited a notably higher ratio of quality-improved reads (average of 38.94%) compared to other pigs (average of 29.22%) (Fig. 4A). We classified NRSs into five categories based on their presence frequencies. We identified a total of 733 core NRSs, 1,502 softcore NRSs, 7,754 shell NRSs, 659 cloud NRSs and 13,183 skeptical NRSs (Table S11). After removing skeptical NRSs, 10,648 NRSs were used for downstream PAV analysis and gene annotation. The PCA plot (Fig. 4B) showed distinct two clusters representing European pigs (Duroc, Landrace and Large White) and other pigs. As expected, European pigs have a noticeably lower number of NRSs than that of other pig breeds due to their closer genetic distance with reference genome (Fig. 4C).

### Identification of the associations between NRSs and pig environmental adaptation

**Fig. 5:**
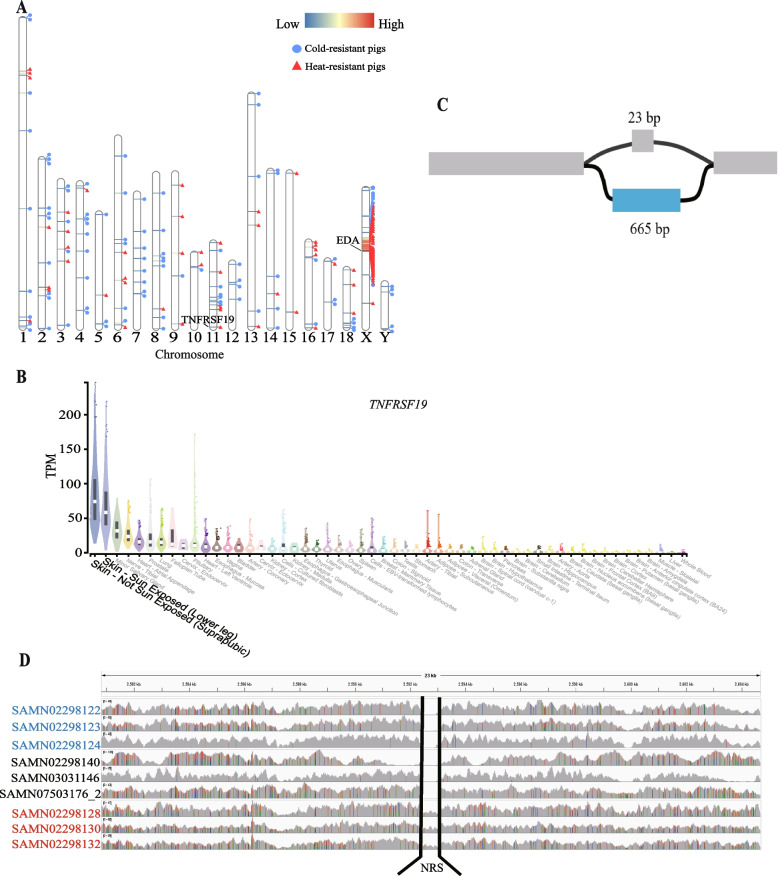
Comparison of frequencies of NRSs in cold-resistant pigs and heat-resistant pigs. **A** Distribution of NRSs with significant high frequencies in cold-resistant pigs and heat-resistant pigs. The horizontal lines in the chromosome represent NRSs, with the red color indicating a higher significant difference of the NRS between cold-resistant pigs and heat-resistant pigs. The blue circles next to the NRS indicate a higher frequency of the NRS in the cold-resistant pigs, while the red triangles indicate a higher frequency of the NRS in the heat-resistant pigs. **B** Graph structure of a 665 bp NRS in *TNFRSF1* that may contribute to the heat tolerance of pigs. **C** Overexpression of *TNFRSF1* in skin in human GTEx data, produced through GTExPortal (https://gtexportal.org/). **D** Comparison of read coverage on the 665 bp NRS of cold-resistant pigs (Min), heat-resistant pigs (Bamaxiang) and three other pigs (Erhualian, Landrace and Bamei). The sample IDs on the left side of the figure are the BioSample IDs of these sample in NCBI. The sample IDs in red font represent Bamaxiang pigs, while the sample IDs in blue font represent Min pigs. The sample IDs in black font are from other pig breeds

We compared the frequency of NRSs in three comparisons (European pigs vs. Asian pigs, cold-resistant pigs vs. heat-resistant pigs, and high-altitude pigs vs. low-altitude pigs) to explore their potential associations with pig environment adaptation (Table 3 and Table S1). We detected a total of 2,496, 193 and 303 NRSs with significantly different frequencies after Bonferroni correction in these three groups, respectively (Table S12, 13, 14). Moreover, 2,280, 102 and 213 NRSs had significantly higher frequencies in Asian pigs, cold-resistant pigs and high-altitude pigs, respectively (Table 3). Interestingly, in the comparison of cold-resistant pigs vs. heat-resistant pigs, 47 PAV-NRSs with significantly different frequencies are located on an 11.6 Mb region of Chromosome X, spanning from 45,231,666 to 56,875,949. The 47 NRSs have an apparently lower *P*-value, and most of which have a higher frequency in heat-resistant pigs (Fig. 5A). This region might have experienced strong selection during their adaptation to tropical environments. There are 59 known protein coding genes located in the 11.6 Mb genomic region according to the annotation of the Ensemble BioMart database (version Ensembl Genes 108) (Table S15). KOBAS annotation shows that the 59 protein coding genes are significantly enriched (with adjusted *P* value < 0.05) in nine GO terms, including basolateral plasma membrane, methylated histone binding, *O*-acyltransferase activity, lipid metabolic process, gamete generation, nucleoplasm, base-excision repair, endonuclease activity and actin cytoskeleton organization.

**Table 3:** Comparison of NRS frequency in different groups identified by Fisher’s exact test

Comparison (Group 1 vs. Group 2)	Samples in Group 1	Samples in Group 2	NRSs with significantly higher frequency in Group 1	NRSs with significantly higher frequency in Group 2
European pigs vs. Asian pigs	40	147	216	2,280
Heat-resistant pigs vs. cold-resistant pigs	31	68	91	102
High-altitude pigs vs. low-altitude pigs	57	43	213	90

European pigs: Duroc, Landrace and Large white. Asian pigs: Bamaxiang, Bamei, Erhualian, Fengjing, Jinhua, Luchuang, Min, Tibetan, Tunchang and Wuzhishan. Heat-resistant pigs: Bamaxiang, Luchuang, Tunchang and Wuzhishan. Cold-resistant pigs: Min and Tibetan. High-altitude pigs: Tibetan. Low-altitude pigs: Erhualian, Fengjing and Jinhua

We performed GO enrichment analysis using the genes that overlapped with the significant NRSs. No significant GO term was found for the comparisons of European pigs vs. Asian pigs and high-altitude pigs vs. low-altitude pigs. Genes overlapped with the 91 NRSs, which were more prevalent in heat-resistant pigs, showed significant enrichment in six GO terms related to skin or hair development (hair follicle development, molting cycle process, hair cycle process, molting cycle, hair cycle and skin epidermis development). Specifically, TNF Receptor Superfamily Member 19 (*TNFRSF19)* and Ectodysplasin A (*EDA*) genes are involved in all six GO terms. The human GTEx dataset also shows that *TNFRSF19* gene is obviously over-expressed in skin (Fig. 5B), implying *TNFRSF19* gene may impact the heat-resistance ability of animals through affecting the development of an animal's skin and fur. However, the *EDA* gene exhibits no specific expression in any tissue in the human GTEx dataset (Fig. S7). We further examined the graph structure of the *TNFRSF19* where the significant NRS occurred. Our investigation revealed that this bubble occurred in the fourth intron of the *TNFRSF19* gene, and consisted of two distinct paths: a reference path positioned at chr11:2,592,452–2,592,475, and an NRS path spanning 665 bp (see the partial graph structure of *TNFRSF19* in Fig. 5C). This NRS shows significantly different frequency between cold-resistant pigs and heat-resistant pigs (*P* = 2.08e-6), presents in 5/68 cold-resistant pigs and 16/32 heat-resistant pigs. The five cold-resistant pigs that have the NRS are all Tibetan pigs. After checking the PAV-NRSs of all 192 samples, we found that only these 21 pigs contain the NRS among all 192 pigs. That is to say, except for 5 Tibetan pigs, only 16 (2 Luchuang pigs, 4 Bamaxiang pigs, 8 TC pigs and 2 Wuzhishan pigs) heat-resistant pigs contains this NRS in our PAV analysis. Furthermore, we used minimap2 to align this 665 bp NRS against both our assembled TC pig genome and the reference genome. The results indicated that this sequence could only be successfully aligned to the TC pig genome. To further verify the PAV of this NRS, we generated a modified reference genome by replacing the 26 bp sequences in the original reference genome with the 665 bp NRS and checked the reads coverage of this region. We randomly selected three cold-resistant pigs, three heat-resistant pigs and three other pigs as control (one for each breed of Erhualian, Landrace and Bamei) and then mapped them to the modified reference genome using all their clean reads. Interestingly, only the three heat-resistant pigs’ reads can cover the NRSs embedded in the reference genome (Fig. 5D). Therefore, we strongly speculated that the 665 bp NRS within *TNFRSF19* might have a big effect on the heat-resistant ability of southern pigs in China.

### Functional annotation of novel predicted genes in NRSs

We conducted repeat annotation for NRSs and found 67.68% (71.20 Mb) of them were repeat sequences (Table S16). The repeat sequences are mainly composed of interspersed repeats (59.70%), including the long interspersed nuclear elements (LINEs, 39.85%), short interspersed nuclear elements (SINEs, 14.17%), Long terminal repeat (LTR, 4.18%), DNA transposons (1.5%). Conversely, the simple repeats and satellites only account for 1.90% and 3.74% of repetitive non-reference bases, respectively.

We predicted a total of 244 high-confident novel genes by removing 3,861 (85.18%) repeat-abundant genes, 314 reference-liked genes (6.93%), and 114 redundant genes (2.51%) from raw 4,533 novel genes predicted in masked NRSs. To verify high-confident predicted novel genes, we examined their expression profile using 145 RNA-seq data. Surprisingly, 88.52% (216 genes) of high-confident novel genes expressed in at least one of the samples. Moreover, 67 of high-confident novel genes expressed in more than 90% of the samples, indicating their potentially important role in the cellular processes, and 68 of them expressed in less than 10% of the samples, implying they were more likely to play role in specific biology functions or be involved in rare or specific biological processes.

We further investigated the functional annotation of high-confident novel genes based on homology alignment by InterProScan, Swissprot and KOBAS tools, and a total of 96, 139 and 133 different entries were annotated by InterProScan, Swissprot and KOBAS respectively (Table S17). The IPR000725 (Olfactory receptor), with 20 annotations, is the most frequently annotated entries in the InterPro database (Fig. S8). We also found that multiple immunity-related entries were annotated, including IPR013106 (Immunoglobulin V-set domain), IPR003597 (Immunoglobulin C1-set), IPR013151 (Immunoglobulin domain). A total of 139 different Swissprot entries in the Swissprot database were annotated by 158 novel genes, among which 75 entries were sourced from human genome. A total of 133 different KEGG entries were annotated by 156 novel genes. Moreover, the KOBAS enrichment analysis revealed high-confident novel genes significantly enriched in 189 terms including 124 GO terms, 24 KEGG pathway terms, 14 KEGG disease terms, and 27 GWAS catalog terms (Table S18). The most significant GO terms are olfactory receptor activity.

## Discussion

The reference genome for pigs, assembled from a European Duroc pig, lacks a significant number of genetic sequences [8, 24]. The identification of these missing sequences, known as NRSs, is crucial to comprehensively understanding the relationship between genetic variations and diverse breed characteristics. In this study, we constructed the pig pangenome graph using two newly assembled pig genomes and 19 publicly available pig genomes. The use of a graph genome is superior to its linear counterpart as it allows for the automatic acquisition of physical coordinates for NRSs during graph construction. Based on the pig pangenome graph, we captured 23,831 NRSs with a cumulative length of 105.16 Mb. Although 72.5 Mb of NRSs had been identified from 12 Eurasian pigs in a previous study, most of them could not be anchored to the reference genome [8]. Another study identified 206 Mb NRNs recently [24], however, the NRNs are interrupted sequence fragments that disrupt the biological integrity of the genomic feature and are detrimental to subsequent gene identification and functional analysis. Therefore, our study recovered NRSs from NRNs and revealed 105.16 Mb of NRSs, all of which have coordinates on the reference genome. These coordinates provide opportunities to further explore the genomic functions of these sequences. The NRSs identified in this study will be an invaluable resource for future research on pig genetics, as they have not been previously studied in depth. Our phylogenetic analysis showed that the genetic difference between Asian pigs, European pigs, and miniature pigs is vast. For example, analysis of the NRNs showed that Chinese native pigs contain more NRNs than European pigs, and the two miniature pigs (EGM and WZS) in an independent phylogenetic clade contain the most NRNs. These NRNs may have important effects on the formation of specific phenotypes in different pigs, particularly for miniature pigs which have been widely used as important animal models in various research areas such as reproduction, transgenesis, and neural development [68].

The independent domestication of pigs in Europe and Asia over 9,000 years ago shaped genetic and phenotypic differences. Chinese pigs often exhibit black skin, higher stress resistance, and lower productivity [69]. The insertion of NRSs may provide insights into the genetic mechanisms underlying different breed features of pigs. Our study demonstrates that NRSs are uniformly distributed within the genomes of pigs, predominantly occurring in non-coding regions. The NRS insertion events are more likely to occur in immune-related genes, which may contribute to the higher stress resistance observed in Chinese local pigs. For instance, the *PI3* (peptidase inhibitor 3) gene encodes a protein that specifically inhibits elastase and exhibits antimicrobial activity against Gram-positive and Gram-negative bacteria, as well as fungal pathogens. The *DEFB1* (defensin beta 1) gene encodes defensin beta 1, an antimicrobial peptide associated with the ability of epithelial surfaces to resist microbial colonization. The protein encoded by the *LTF* (lactotransferrin) gene displays a broad range of functions, including the regulation of iron homeostasis, host defense against diverse microbial infections, anti-inflammatory activity, regulation of cellular growth and differentiation, and protection against cancer development and metastasis.

Our analysis of PAV-NRSs has revealed significant genetic differences between Asian and European pigs, indicating substantial population divergence. Since the reference genome used in this study was derived from a European pig, it is expected that the majority of the significantly different NRSs would have higher frequencies in Asian pigs. Interestingly, the genes affected by these NRSs did not show significant enrichment in any GO terms, suggesting that these differences are not primarily concentrated in any specific functional biological processes. However, our analysis did identify several significant NRSs associated with pig environmental adaptation. Through a comparative analysis of NRSs frequency differences between cold-resistant and heat-resistant pigs, we observed an enrichment of 47 significant NRSs within an 11.6 Mb interval (from 45,231,666 to 56,875,949) on the X chromosome. We found that the repetitive sequence content in this region accounted for 69.23%, surpassing the average repetitive sequence proportion in the pig reference genome (44.79%). Moreover, we discovered that the NRS density, which represents the number of NRSs per Mb, within this 11.6 Mb interval (15.60) was notably higher than the NRS density on the X chromosome (12.14) and the autosomes (10.19). Additionally, we observed significantly higher frequencies of NRSs in *EDA* and *TNFRSF19* genes in tropical and subtropical pigs. The *EDA* gene is involved in the formation and development of skin appendages in vertebrates, such as keratinocytes, hair follicles, and sweat glands [70]. Additionally, we identified a 665 bp NRS embedded in *TNFRSF19* in heat-resistant pigs. The *TNFRSF19* gene encodes TNF-receptor proteins and plays an important role in hair follicle development, skin development, and epithelial cell development [71, 72]. The human GTEx data shown that the *TNFRSF19* gene is highly expressed in skin [73]. Moreover, the *TNFRSF19* gene has also been reported to be associated with intramuscular fat content and fatty acid composition traits in pigs and there are three QTLs (intramuscular fat content, oleic content and linoleic acid content) were near the *TNFRSF19* gene in pig reference genome [74]. These results suggest that NRSs may play an important role in the adaptation of pigs to different environments and highlight the need to consider these variations when exploring the genetic mechanisms underlying certain traits.

The resequencing data revealed that when considering NRSs, a greater number of reads aligned to the appropriate locations. Approximately one-third of low-mapping-quality reads could be successfully mapped to the NRSs with high mapping quality, indicating the presence of numerous suboptimal alignments when relying solely on the reference genome. Suboptimal alignments can be attributed to the frequent presence of false-positive alignments in BWA when default parameters are used [16]. The mapping procedure based on the graph genome is known to be time-consuming and memory-intensive [3]. Therefore, it may be more appropriate to map reads with low mapping quality to the NRSs, as this approach could also improve mapping accuracy.

## Conclusions

In conclusion, this study demonstrates the effectiveness of the graph genome approach in identifying novel genetic sequences in pigs and provides new insights into the genetic diversity and environmental adaptation of pigs. The graph genome approach employed in this investigation holds promise for enhancing reference genomes and uncovering previously overlooked genetic variations in other species as well. Furthermore, the NRSs identified in this study have the potential to significantly impact the development of breed-specific phenotypic traits and can prove valuable in future endeavors related to pig breeding and genetic engineering.

### Supplementary Information

**Additional file 1: Fig. S1.** Assembly contiguity shown as a NGx plot. Contigs of Meishan (MS) pig and Ningxiang(NX) pig are included for comparison. **Fig. S2.** Phylogenetic tree of the *Sus scrofa 11.1* assembly and 20 other pig assemblies. **Fig. S3.** Violin plot illustrating the cumulative length of non-reference sequences in males and female pigs. **Fig. S4.** Distribution of NRSs across the chromosomes. The blue squares represent cNRSs, while the red squares represent pNRSs. **Fig. S5.** Proportions of different QTL classes where cNRSs occurred. **Fig. S6.** Proportions of different QTL classes where pNRSs occurred. **Fig. S7.** The expression of EDA in different tissues in human GTEx data, produced through GTExPortal (https://gtexportal.org/). **Fig. S8.** Top 10 significant most frequently annotated entries in InterPro database.

**Additional file 2: Table S1.** The information of samples used for population-based NRS analysis. **Table S2.** he enrichment analysis of genes affected by deleterious variants for SWT and TC pig. **Table S3.** rchored ratio of 15 pig assemblies. **Table S4.** The distance between *Sus scrofa* 11.1 and other 20 assemblies calculated using Mash. **Table S5.** The information of Non-reference nodes (NRNs) in 20 assemblies. **Table S6.** The information of NRSs in each step of filtering. **Table S7.** GO enrichment analysis of genes whose CDS regions were affected by pNRS insertion events. **Table S8.** Enrichment analysis of QTLs where the pNRS insertion occurred. **Table S9.** Enrichment analysis of QTLs where the cNRS insertion occurred. **Table S10.** The information of low-quality reads mapping to NRSs. **Table S11.** The classification of 23,831 NRSs. **Table S12.** Fisher’s exact test for the difference of NRS frequencies between Asian pigs and European pigs. **Table S13.** Fisher’s exact test for the difference of NRS frequencies between cold-resistant pigs and heat-resistant pigs. **Table S14.** Fisher’s exact test for the difference of NRS frequencies between high-altitude pigs and low-altitude pigs. **Table S15.** Genes residing in the 11.6 Mb region of Chromosome X (ChrX: 45,231,666:56,875,949) enriched by 47 significantly different NRS between cold-resistant pigs and heat-resistant pigs. **Table S16.** Repeats annotated by RepeatMasker. **Table S17.** Novel genes annotated by Interproscan, Swissprot and KOBAS. **Table S18.** Significant terms (corrected *P* value < 0.05) of functional enrichment analysis of novel genes using KOBAS.

## Data Availability

The whole genome sequencing datasets analyzed during the current study are available in the NCBI Sequence Read Archive (http://www.ncbi.nlm.nih.gov/sra/) and under project PRJNA311523, PRJNA398176, PRJNA213179, PRJNA550237, PRJNA751703, PRJNA260763, PRJNA488327, PRJEB9922, PRJNA369600, PRJNA343658, PRJNA378496, PANLAB_ASF, PRJNA305081, PRJNA671763, PRJNA186497, PRJNA438040. The RNA-seq dataset are available in the NCBI Gene Expression Omnibus (https://www.ncbi.nlm.nih.gov/geo/) and CNGB Nucleotide Sequence Archive in (https://db.cngb.org/cnsa/) under project GSE77776 and CNP0001361. All raw sequencing data generated in this study have been submitted to the Genome Sequence Archive in China National Center for Bioinformation (https://ngdc.cncb.ac.cn/bioproject/) under accession number PRJCA017882. The assemblies generated in this study is available from https://figshare.com/s/155e92372e5e8e7b4e0a. Additionally, other relevant supplementary data is available from https://figshare.com/s/adc945fbfe4f3f8ea423.

## Abbreviations

cNRS: Complete non-reference sequence
NRN: Non-reference node
NRS: Non-reference sequence
pNRS: Partial non-reference sequence
PAV: Presence/absence variation
PAV-NRSs: Presence/absence variation of NRSs
PCA: Principal component analysis
SNP: Single nucleotide polymorphism
WGS: Whole genome sequencing
