# Supplementary material for: Pig pangenome graph reveals functional features of non-reference sequences
Source: J Anim Sci Biotechnol. 2024 Feb 22;15:32. doi: 10.1186/s40104-023-00984-4 (PMC10882747; doi:10.1186/s40104-023-00984-4)
Supplement: Supplementary file 1 — Additional file 1: Fig. S1. Assembly contiguity shown as a NGx plot. Contigs of Meishan (MS) pig and Ningxiang(NX) pig are included for comparison. Fig. S2. Phylogenetic tree of the Sus scrofa 11.1 assembly and 20 other pig assemblies. Fig. S3. Violin plot illustrating the cumulative length of non-reference sequences in males and female pigs. Fig. S4. Distribution of NRSs across the chromosomes. The blue squares represent cNRSs, while the red squares represent pNRSs. Fig. S5. Proportions of different QTL classes where cNRSs occurred. Fig. S6. Proportions of different QTL classes where pNRSs occurred. Fig. S7. The expression of EDA in different tissues in human GTEx data, produced through GTExPortal (https://gtexportal.org/). Fig. S8. Top 10 significant most frequently annotated entries in InterPro database. [file 40104_2023_984_MOESM1_ESM.docx]

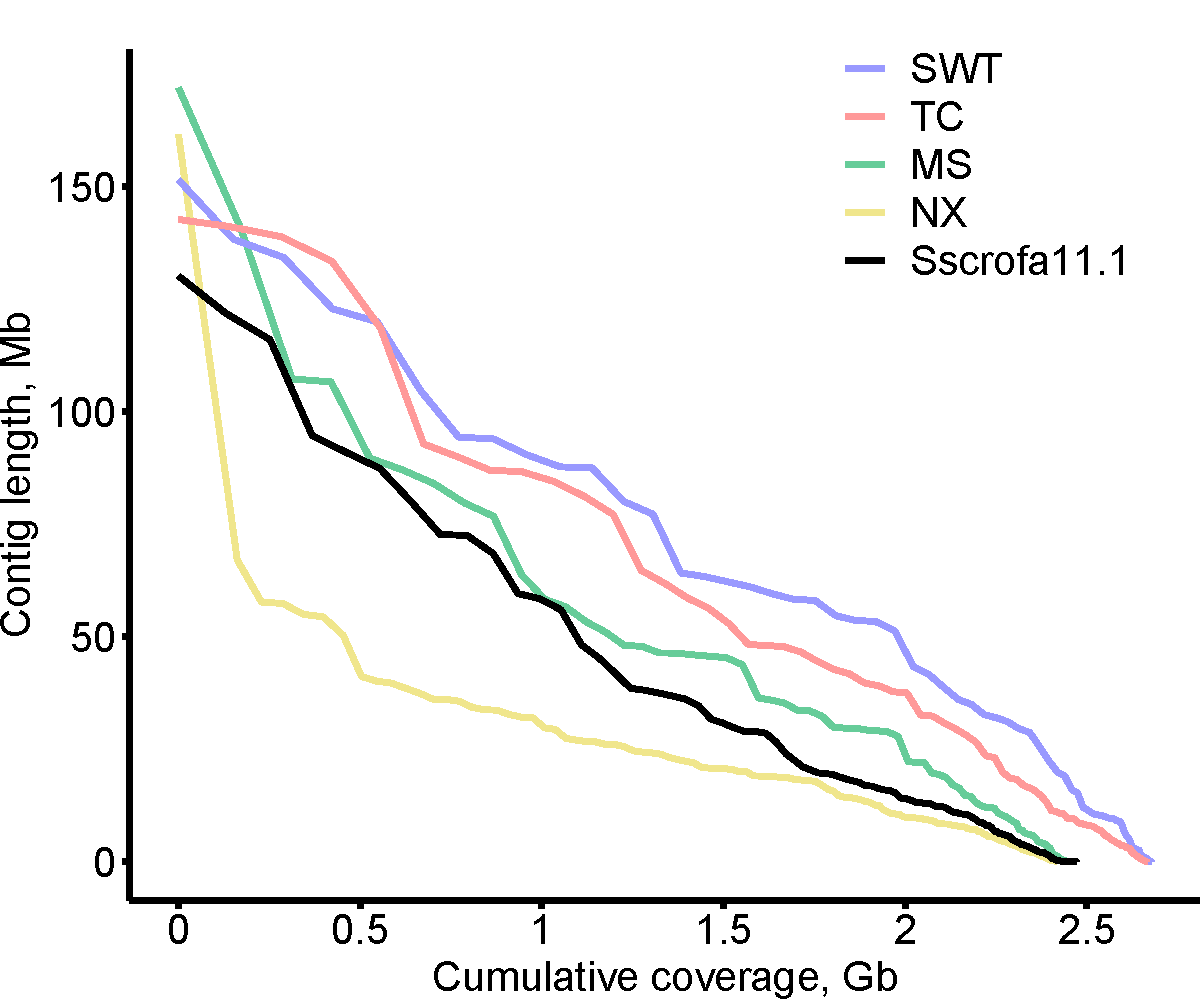


**Fig. S1** Assembly contiguity shown as a NGx plot. Contigs of Meishan (MS) pig and Ningxiang(NX) pig are included for comparison


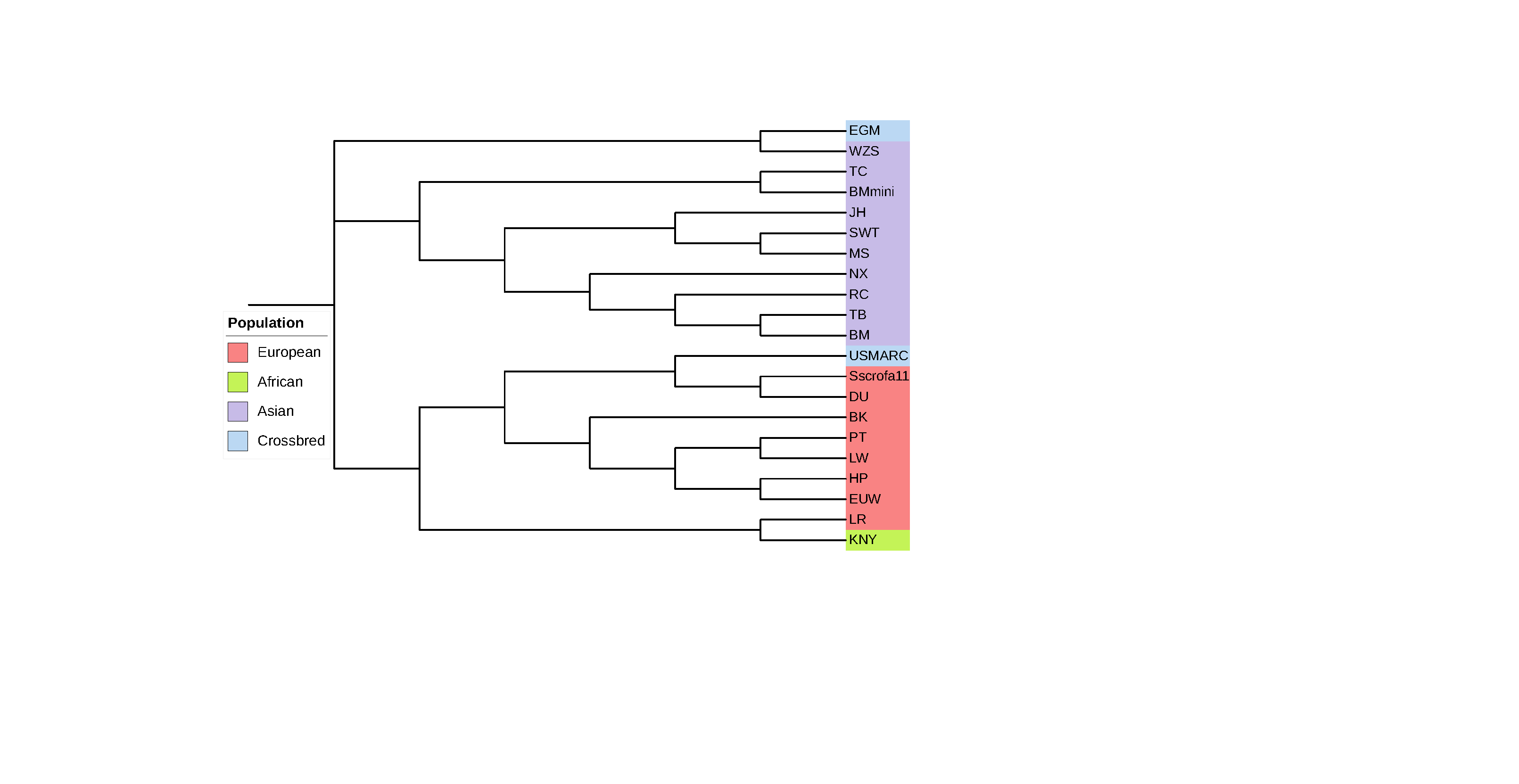


**Fig. S2** Phylogenetic tree of the *Sus scrofa 11.1* assembly and 20 other pig assemblies


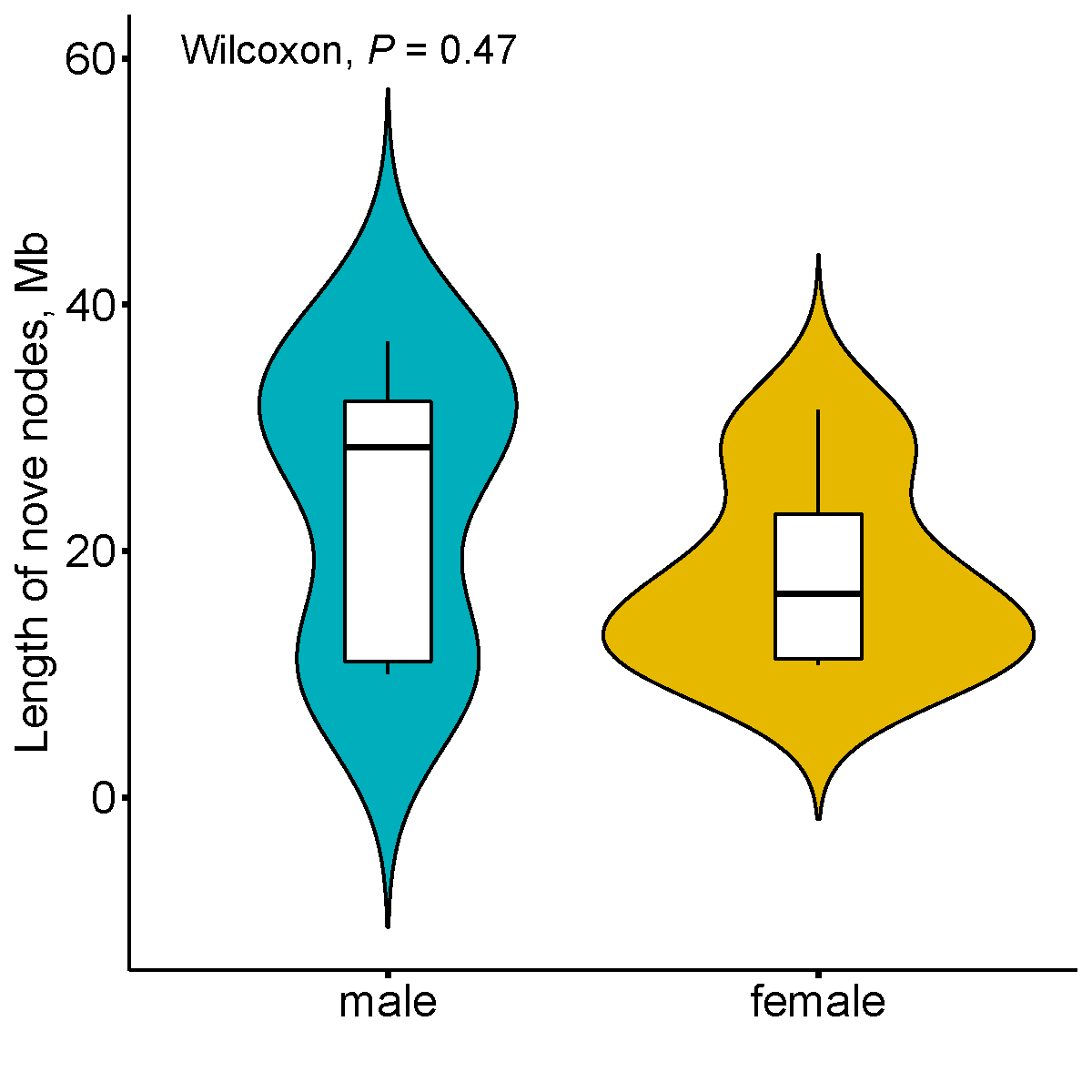


**Fig. S3** Violin plot illustrating the cumulative length of non-reference sequences in males and female pigs
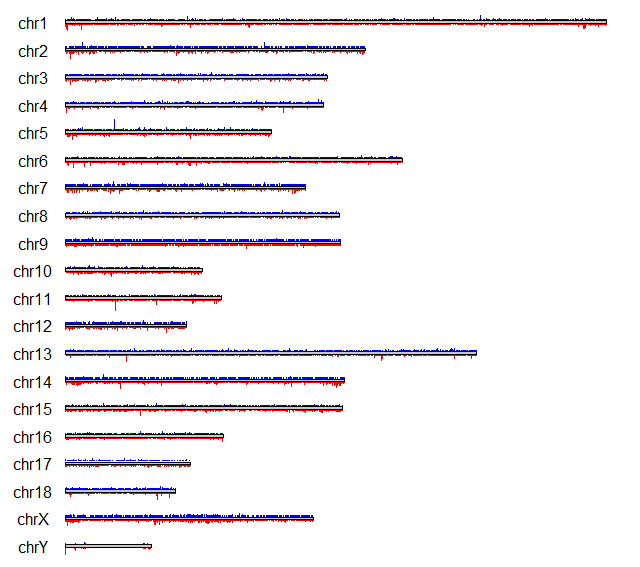


**Fig. S4** Distribution of NRSs across the chromosomes. The blue squares represent cNRSs, while the red squares represent pNRSs


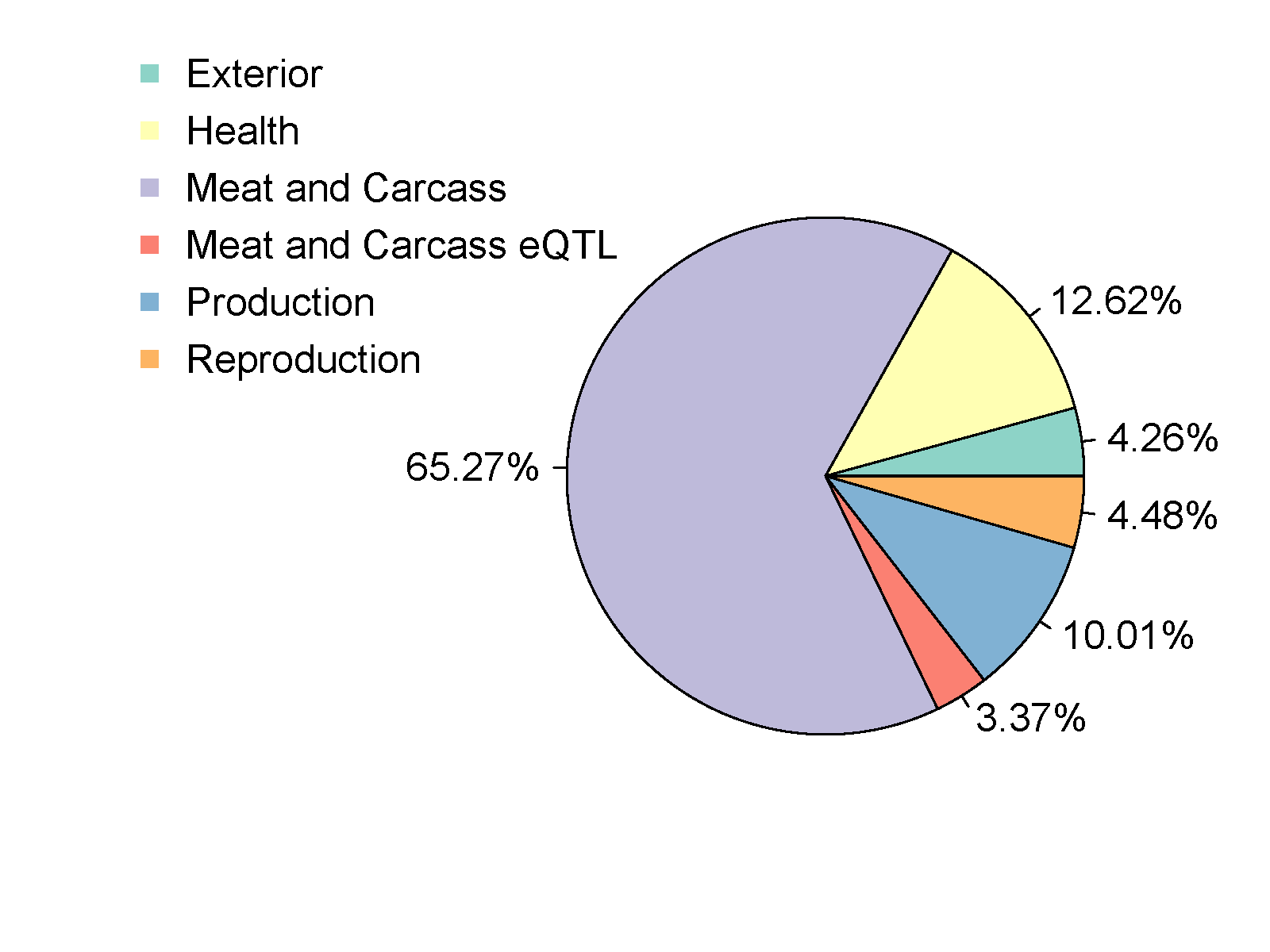


**Fig. S5** Proportions of different QTL classes where cNRSs occurred


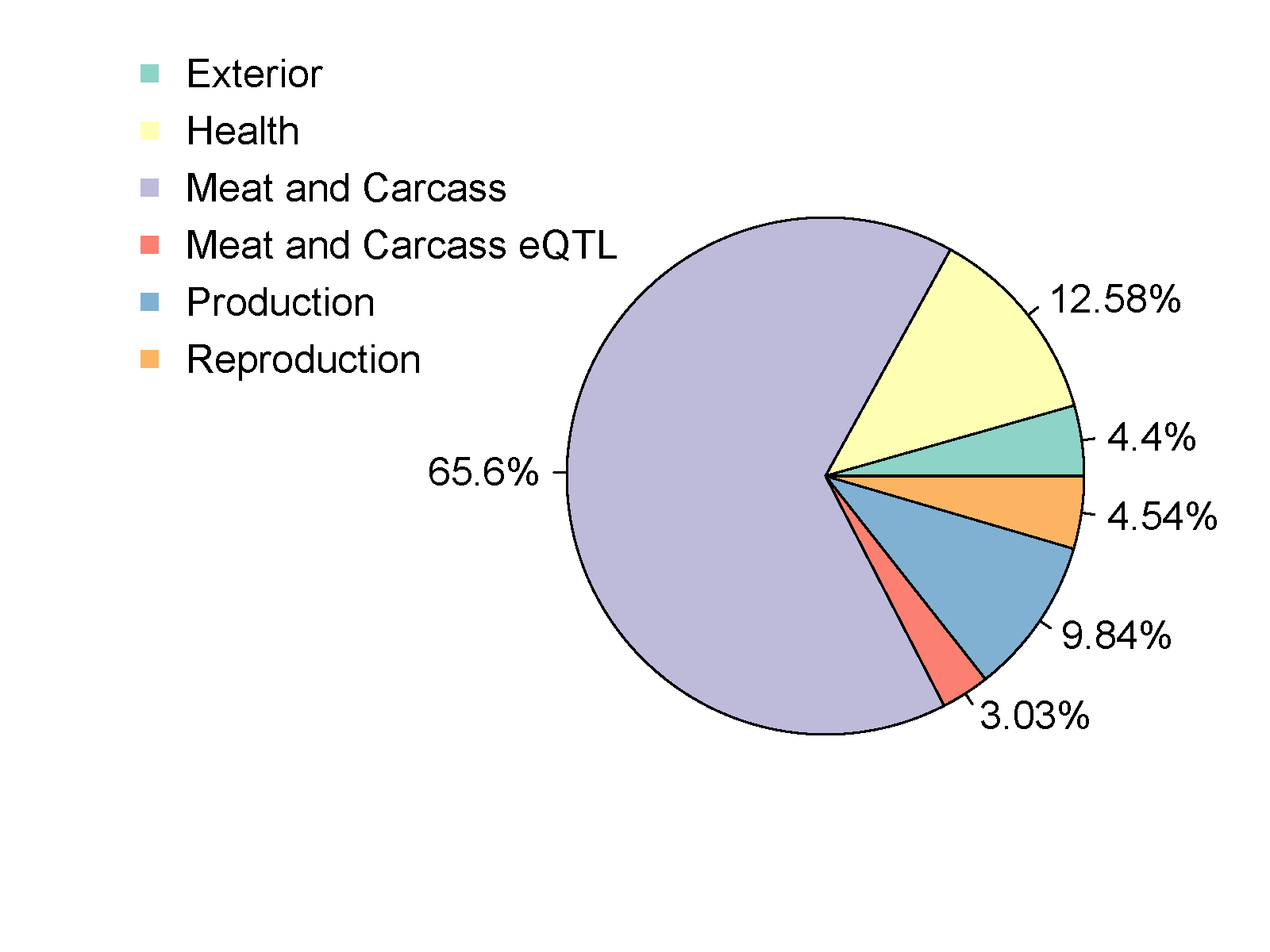


**Fig. S6** Proportions of different QTL classes where pNRSs occurred
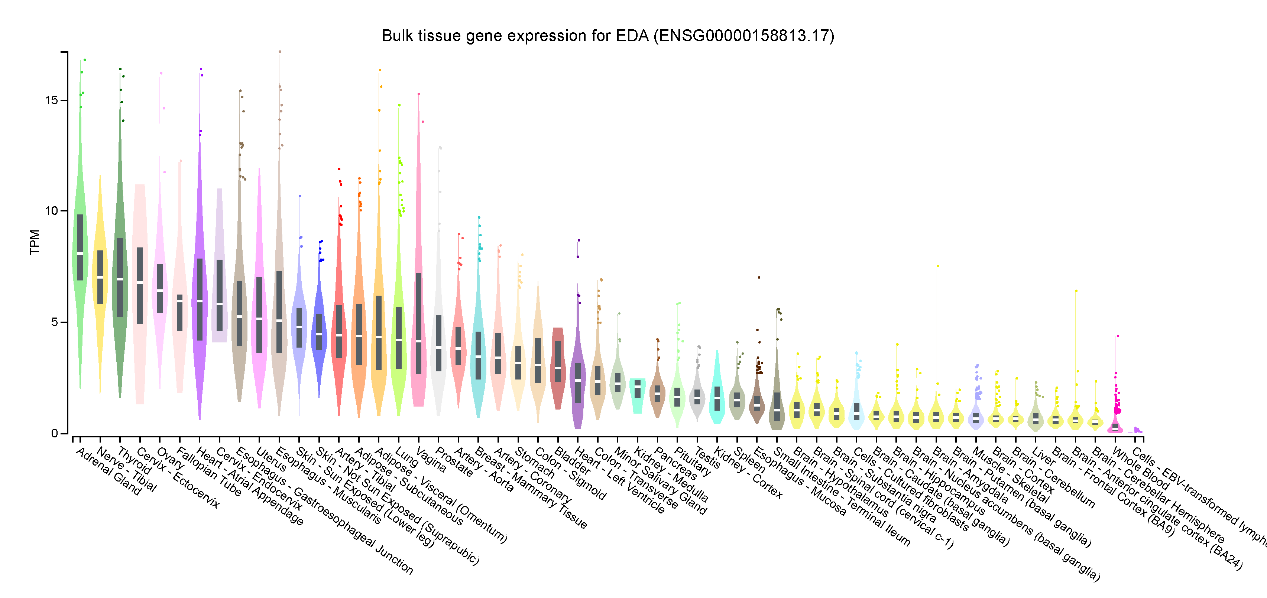


**Fig. S7** The expression of EDA in different tissues in human Gtex data, produced through GTExPortal (https://gtexportal.org/)


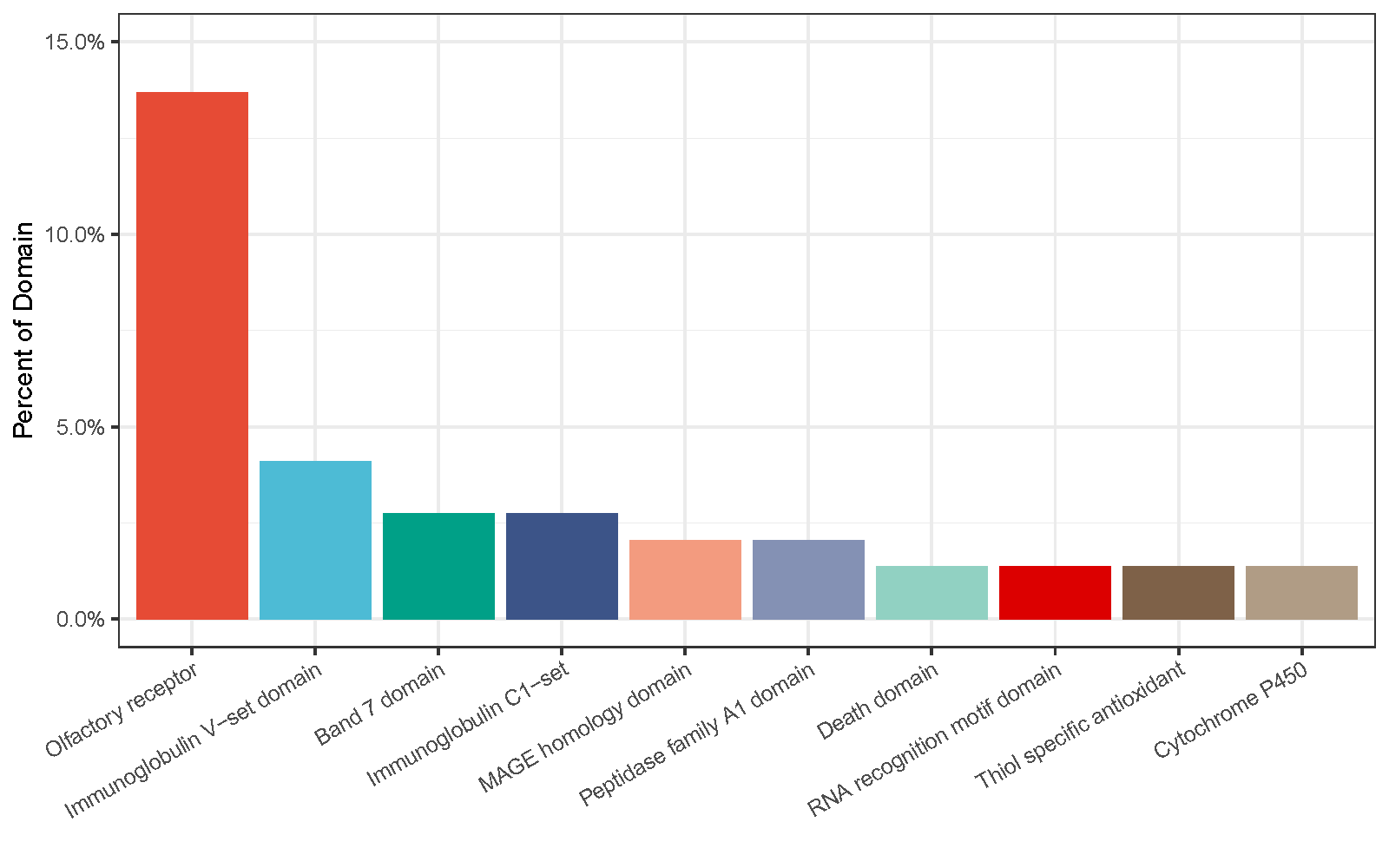


**Fig. S8** Top 10 significant most frequently annotated entries in InterPro database
